# Cervical Pyogenic Spondylitis: A Comprehensive Review of Diagnosis and Treatment Strategy

**DOI:** 10.3390/jcm14103519

**Published:** 2025-05-17

**Authors:** Chae-Gwan Kong, Sung-Kyu Kim, Jong-Beom Park

**Affiliations:** 1 Department of Orthopaedic Surgery, Uijeongbu St. Mary’s Hospital, The Catholic University of Korea College of Medicine, Uijeongbu 11765, Republic of Korea; gongjae@catholic.ac.kr; 2 Department of Orthopaedic Surgery, Chonnam National University Medical College & Hospital, Gwangju 61469, Republic of Korea; bonjourksk@hanmail.net

**Keywords:** cervical spine, pyogenic spondylitis, surgical intervention, interbody graft, spinal instrumentation, antibiotic therapy, diagnosis

## Abstract

Cervical pyogenic spondylitis (CPS) is a rare but serious spinal infection with a high risk of neurological compromise due to the cervical spine’s narrow canal and proximity to critical neurovascular structures. Early diagnosis relies on a high index of suspicion supported by MRI, inflammatory markers, blood cultures, and tissue biopsy. Empirical intravenous antibiotics remain the cornerstone of initial treatment, followed by pathogen-specific therapy. Surgical intervention is indicated in cases of neurological deterioration, spinal instability, or failure of conservative management. Anterior approaches, including anterior cervical discectomy and fusion (ACDF) and anterior cervical corpectomy and fusion (ACCF), are widely used, with anterior plating providing biomechanical advantages in select cases. Posterior or combined anterior–posterior approaches are recommended in multilevel disease, deformity, or posterior element involvement. Graft selection—typically autograft or titanium/PEEK cages—must consider infection severity and biomechanical demands. Challenges in CPS management include optimal debridement extent, graft choice in infected environments, the standardization of antibiotic protocols, and the prevention of recurrence. This narrative review synthesizes the cervical-spine-specific literature on diagnosis, treatment strategies, surgical techniques, and postoperative care and proposes the following practical clinical guidance: (1) early MRI for timely diagnosis, (2) prompt surgical intervention in patients with neurological deficits or mechanical instability, and (3) individualized graft selection based on infection severity and bone quality.

## 1. Introduction

**Figure 1:**
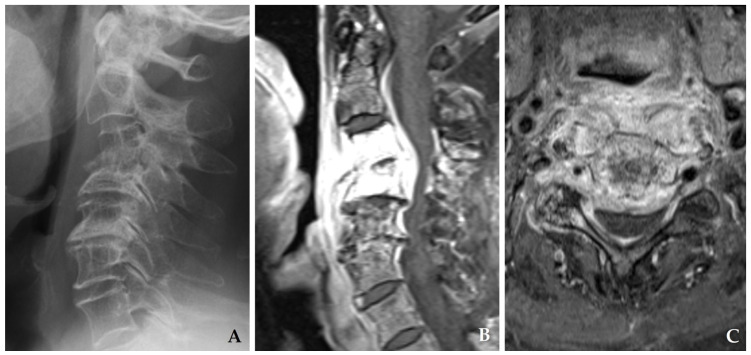
Plain radiograph (**A**) and magnetic resonance imaging (**B**,**C**) demonstrate destructive changes in the C3–C4 vertebral bodies with kyphotic deformity accompanied by retropharyngeal and epidural abscesses compressing the spinal cord.

Cervical pyogenic spondylitis (CPS) is an uncommon but clinically significant subtype of vertebral osteomyelitis, accounting for 3–6% of all pyogenic spinal infections [1,2,3]. Despite its rarity, CPS poses a substantial threat due to the cervical spine’s close relationship with the spinal cord and brainstem. Even minor infections in this region may lead to rapid neurological deterioration or death, highlighting the importance of prompt diagnosis and management [4] (Figure 1).

The pathogenesis of CPS is predominantly hematogenous, with bacterial dissemination from distant infectious sources such as infective endocarditis, urinary tract infections, pulmonary infections, and cutaneous or soft tissue abscesses. Established risk factors include diabetes mellitus, immunosuppression, intravenous drug use, malignancy, and recent invasive spinal or systemic procedures [5,6]. Despite advances in imaging techniques, early diagnosis remains challenging. The clinical presentation is often nonspecific, commonly including axial neck pain, fever, and paraspinal tenderness, and is frequently misattributed to more prevalent conditions such as cervical spondylosis or spinal neoplasms. This diagnostic ambiguity contributes to delays in definitive management, thereby increasing the risk of serious complications, such as epidural abscess formation, vertebral body destruction, and irreversible neurological injury.

Although multiple reviews have addressed pyogenic spondylitis in general, most focus on thoracolumbar involvement and lack cervical-spine-specific analysis [7,8,9,10,11]. Given the unique anatomical constraints, vascular supply, and neurological vulnerability of the cervical region, CPS presents distinct diagnostic and therapeutic challenges. Furthermore, there is a notable absence of prospective studies or high-level evidence tailored to CPS. Current practices are largely extrapolated from the thoracic and lumbar spine literature, which may not be directly applicable. This review differs from the prior literature by focusing exclusively on cervical spondylitis by critically synthesizing surgical strategies, instrumentation considerations, graft materials, and diagnostic nuances unique to the cervical spine. This review aims to fill the gap by providing a comprehensive and practical clinical guide for spine specialists dealing with CPS.

## 2. Materials and Methods

This article is a narrative review focused on the diagnosis and management of CPS. Given the rarity of CPS and the highly heterogeneous nature of the existing literature, a systematic review was deemed inappropriate for this study. Most available studies are retrospective case series, case reports, or single-institution observational analyses with small sample sizes and inconsistent outcome reporting. Moreover, there is a marked absence of high-quality randomized controlled trials (RCTs) or standardized diagnostic and therapeutic protocols specific to CPS. Studies vary widely in inclusion criteria, surgical techniques, graft choices, definitions of treatment success, and follow-up durations, which limits direct comparability or the meta-analytic pooling of data.

While a comprehensive literature search was conducted to identify all relevant studies, the lack of uniform methodology and data standardization precluded a systematic synthesis or evidence grading. Therefore, a narrative review format was selected to allow a critical appraisal of the literature, the integration of cervical-specific clinical insights, and the structured interpretation of evolving practices. This approach is appropriate and necessary for rare and under-researched conditions like CPS, where expert opinion and contextual analysis remain essential to guide clinical decision making.

A literature search was conducted using PubMed, Scopus, and Web of Science up to March 2025. Search terms included “cervical pyogenic spondylitis”, “cervical spinal infection”, “anterior cervical surgery”, “instrumentation”, “interbody graft”, and related terms. Only English-language articles published in peer-reviewed journals were included. Preference was given to studies reporting clinical outcomes, diagnostic strategies, surgical techniques, or cervical-specific data. Case reports with fewer than five patients and studies unrelated to cervical infection were excluded.

This review emphasizes cervical-specific microbiology, surgical indications, fusion techniques, and postoperative considerations, with particular attention to the role of instrumentation, graft materials, and complications in infected environments.

### 2.1. Inclusion Criteria

Studies were included if they met the following criteria:

- Peer-reviewed full-text articles published in English;
- Focused specifically on CPS rather than generalized spondylodiscitis or thoracolumbar infection;
- Reported clinical data including diagnosis, treatment strategy (conservative and/or surgical), surgical technique, outcomes, or complications;
- Included at least five or more patients diagnosed with CPS;
- Provided sufficient detail on surgical approach (e.g., ACDF, ACCF, posterior, or combined), graft selection, instrumentation use, and/or follow-up results.

### 2.2. Exclusion Criteria

The following were excluded:

- Case reports or case series involving fewer than five patients;
- Studies that addressed only thoracic or lumbar spondylodiscitis without cervical data;
- Articles that were not published in English and had no available full-text translation;
- Review articles, editorials, expert opinions, or guidelines that lacked original CPS-specific clinical data.

## 3. Pathophysiology and Microbiology

CPS leads to rapid vertebral and neural compromise due to the cervical spine’s structural fragility and proximity to critical neurological pathways. Infection typically originates from the hematogenous seeding of the vertebral endplates, but the subsequent pathophysiology involves a complex interplay of host inflammatory response, structural degradation, and microbial virulence.

### 3.1. Disease Progression and Pathophysiological Mechanisms of CPS

The progression of CPS can be conceptualized in four overlapping stages, now understood through updated molecular and immunological mechanisms [7,8,9,10,11].

(1) Bacterial Inoculation and Early Inflammation

Pathogens reach the cervical spine via bloodstream dissemination from distant sources. The anterior endplate’s rich vascularity often serves as the entry site.

(2) Cytokine-Mediated Inflammatory Cascade

The activation of the innate immune system triggers a release of pro-inflammatory cytokines (e.g., TNF-α, IL-1β, IL-6), matrix metalloproteinases (MMPs), and reactive oxygen species. This cascade promotes the following:

- Vascular hyperpermeability and bone marrow edema;
- Disc degeneration and endplate erosion;
- Early paraspinal soft tissue inflammation.

(3) Structural Collapse and Vascular Disruption

The infection extends into the intervertebral discs and vertebral endplates, causing the infection to spread across the disc–vertebral complex, leading to cortical bone destruction, vertebral body collapse, and kyphotic deformity. Impaired microvascular supply exacerbates ischemia, promoting necrosis and mechanical instability.

(4) Abscess Formation and Neural Injury

Accumulated pus in the epidural or retropharyngeal space results in spinal cord compression. Neuronal injury arises from both direct pressure and cytokine-mediated neurotoxicity, necessitating urgent decompression. This updated framework highlights that neurological decline in CPS is not merely mechanical, but also a consequence of host immune dysregulation.

### 3.2. Microbiology: Focus on Cervical-Spine-Specific Trends

Staphylococcus aureus (including MRSA) remains the most common organism, often associated with aggressive disease and early abscess formation. In the cervical region, pathogens such as *Streptococcus viridans*, *Pseudomonas aeruginosa*, and anaerobes from oropharyngeal flora are more frequently implicated than in thoracolumbar cases due to anatomical proximity [7,8,9,10,11,12].

Notably, *Mycobacterium tuberculosis* should be considered in endemic regions or immunocompromised hosts. Fungal infections (e.g., *Candida*, *Aspergillus*) may present subtly and require specialized stains or PCR for detection. Increasing rates of culture-negative CPS, possibly due to prior antibiotic use, warrant the use of broad-range PCR and histopathology to detect elusive organisms. Table 1 provides a comprehensive summary of causative organisms and clinical features and associations.

## 4. Clinical Presentations

The clinical manifestations of CPS are highly variable and often nonspecific in early stages. However, certain features distinguish CPS from thoracic or lumbar infections, reflecting the unique anatomy and neurovascular density of the cervical spine [13,14].

### 4.1. Common and High-Priority Presenting Symptoms

(1) Axial neck pain is the most frequent early symptom, often localized and progressive.
(2) Neurological deficits are more common and severe in CPS than in thoracolumbar infections due to the narrower spinal canal and proximity to the brainstem.
  - Early signs include upper limb paresthesia, hand clumsiness, or gait imbalance.
  - Delayed diagnosis may lead to spastic quadriparesis or respiratory compromise in high cervical lesions (C1–C4).
(3) Fever may be absent in up to 50% of cases, especially in immunocompromised or elderly patients.
(4) Dysphagia, hoarseness, or anterior neck swelling may occur with prevertebral or retropharyngeal abscess extension, which is unique to the cervical region.
(5) Neck stiffness may mimic meningitis but should raise suspicion when focal tenderness or elevated inflammatory markers are present.

### 4.2. Atypical or Delayed Presentations

(1) Referred shoulder pain, occipital headache (high cervical involvement).
(2) Drop attacks, vertigo, or syncope (suggesting vertebral artery compromise).
(3) Elderly patients may present with delirium or acute functional decline without overt infection signs.

These features warrant a high index of suspicion, particularly when radiographic findings lag behind clinical symptoms.

Table 2 provides a comparative summary of the clinical features between cervical and thoracolumbar pyogenic spondylitis [4,15,16].

### 4.3. Clinical Course

The clinical course of CPS is highly variable and depends on several key factors. This section emphasizes that timing matters; even subtle delays in diagnosis can have profound consequences in CPS [4,16].

(1) Pathogen virulence:

Infections caused by highly virulent organisms, such as methicillin-resistant *Staphylococcus aureus* (MRSA) or Gram-negative bacilli, are often associated with rapid progression, early epidural abscess formation, and vertebral collapse. These cases frequently require urgent surgical intervention.

(2) Host immune status:

Immunocompromised patients, including those with diabetes, malignancy, or chronic renal failure, may present with subtle or delayed symptoms, leading to diagnostic delays and worse outcomes.

(3) Timing of diagnosis and treatment initiation:

Early recognition and prompt initiation of pathogen-targeted antibiotic therapy—with or without surgical intervention—are the most critical factors for preventing permanent neurological deficits and structural instability.

(4) Neurological trajectory:

Once neurological symptoms emerge, deterioration can occur abruptly and irreversibly, especially in high cervical lesions (C1–C4). Therefore, rapid imaging and early surgical consultation are essential in any patient with cervical pain and neurologic change.

## 5. Diagnostic Modalities

**Figure 2:**
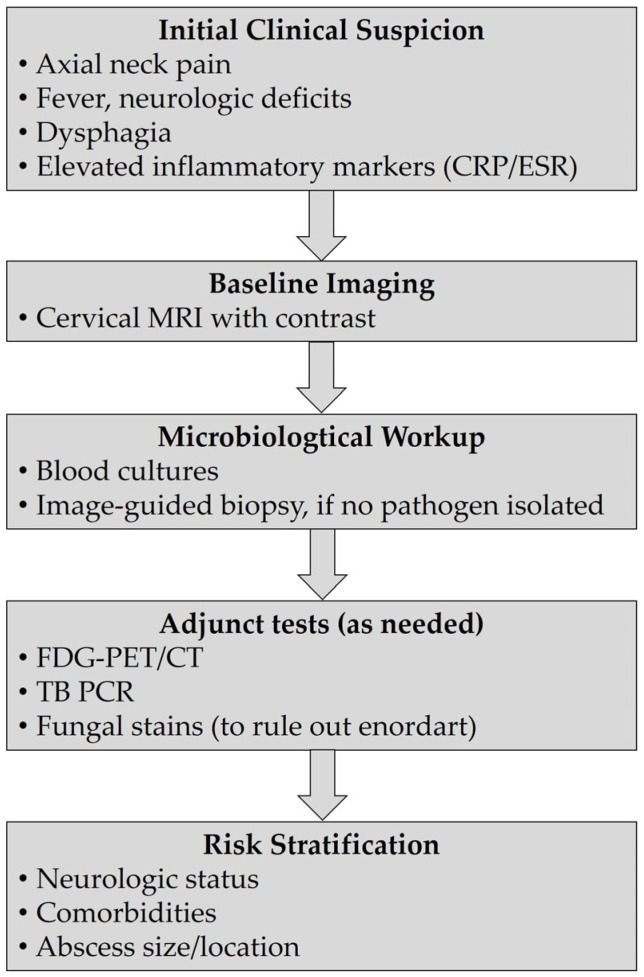
Diagnostic algorithm for CPS.

The diagnosis of CPS necessitates a comprehensive multimodal approach tailored to the cervical spine’s unique anatomical and clinical characteristics. Timely and accurate diagnosis is essential to prevent serious complications, including irreversible neurological injury, spinal instability, and systemic sepsis (Figure 2).

### 5.1. Clinical Evaluation

Accurate diagnosis begins with a targeted clinical history and thorough physical examination, emphasizing cervical-spine-specific findings [17].

(1) History Taking

Key presenting symptoms include persistent cervical pain, fever, and neurological deficits. A detailed medical history should evaluate predisposing factors such as immunosuppression, diabetes mellitus, intravenous drug use, prior infections, and recent cervical instrumentation. Due to the nonspecific nature of early symptoms, CPS is frequently misdiagnosed as meningitis, cervical spondylosis, or metastatic spinal tumors. Atypical presentations can result in diagnostic delays. Persistent or unexplained cervical pain, especially when accompanied by neurological signs, warrants a high index of suspicion and repeated clinical reassessment.

(2) Physical Examination

The physical exam should include palpation for focal tenderness along the cervical spine, spinal alignment or deformity assessment, and a comprehensive neurological examination. This should encompass motor strength, sensory function, deep tendon reflexes, gait analysis, and evaluation for signs of myelopathy or radiculopathy. Given the cervical spine’s proximity to the spinal cord, the early recognition of subtle neurological deficits is imperative to prevent clinical deterioration.

### 5.2. Laboratory Investigations

Laboratory studies are vital for detecting systemic inflammation, identifying causative pathogens, and guiding antimicrobial therapy [17].

(1) Inflammatory Markers

Elevated C-reactive protein (CRP) and erythrocyte sedimentation rate (ESR) are common and serve as valuable indicators of disease activity and therapeutic response. CRP is especially sensitive during the acute phase due to its rapid kinetics.

(2) Leukocyte Count

Leukocytosis may be absent, particularly in immunocompromised or chronically infected patients. The white blood cell count should be interpreted in conjunction with clinical and other laboratory data.

(3) Blood Cultures

Positive blood cultures are found in approximately 30–60% of CPS cases, particularly when obtained during febrile episodes. Cultures should be drawn before antibiotic administration to optimize yield and enable pathogen-specific therapy.

(4) Biopsy and Tissue Culture Modalities

CT-guided percutaneous biopsy offers high diagnostic accuracy, particularly in culture-negative or subacute cases. Whenever possible, tissue sampling should be performed prior to initiating empirical antibiotics to maximize the chances of pathogen identification and support targeted antimicrobial treatment [18,19].

Accurate microbiological diagnosis is critical for targeted therapy. In addition to traditional culture, advanced microbiological techniques are increasingly necessary in CPS:

- Sample Number and Timing: At least two to three specimens should be collected during image-guided biopsy or surgery before antibiotic initiation.
- Mycobacterial and Fungal Cultures: These are particularly important in immunocompromised patients or in endemic TB areas. Mycobacterium tuberculosis often presents with indolent symptoms and may require prolonged culture (up to 8 weeks).
- Polymerase Chain Reaction (PCR): This is highly beneficial in cases where antibiotics were administered before sampling, as it can detect bacterial DNA even in culture-negative scenarios. PCR assays for TB, *Staphylococcus aureus*, or broad-spectrum 16S rRNA can guide early decisions.
- Histopathology: This should accompany culture to evaluate for granulomatous inflammation, necrosis, or pyogenic changes.

### 5.3. Imaging Studies

Imaging is central to confirming the diagnosis, determining disease extent, planning surgical intervention, and monitoring treatment response [20,21].

(1) Magnetic Resonance Imaging (MRI)

MRI remains the primary modality for the initial diagnosis of CPS, but it has important limitations in post-treatment follow-up. Inflammatory changes on MRI, including vertebral enhancement, disc signal alteration, and prevertebral soft tissue edema, may persist for weeks to months despite clinical resolution. This can complicate decisions regarding treatment duration or the need for further intervention.

Several key points should be considered in follow-up imaging:

- Persistent MRI abnormalities do not necessarily indicate treatment failure. MRI should be interpreted in conjunction with the patient’s clinical course, neurological status, and laboratory markers (CRP, ESR).
- Residual contrast enhancement may reflect granulation tissue rather than active infection.
- In postoperative patients, metal artifact and reactive surgical changes may further obscure interpretation.
- MRI is not reliable for distinguishing sterile inflammation from residual infection; FDG-PET/CT may be useful in this setting.

Therefore, imaging should not be the sole determinant for treatment duration or surgical decision making. A multimodal approach integrating imaging, clinical improvement, and laboratory normalization is essential for safe and effective CPS monitoring.

Differentiating pyogenic vs. tuberculous spondylitis on MRI is essential, especially in regions endemic to tuberculosis. Pyogenic spondylitis typically demonstrates the following:

- Homogeneous vertebral body enhancement;
- Early and aggressive disc space involvement;
- Rim-enhancing abscesses (in ~64% of cases).

By contrast, tuberculous spondylitis often shows the following:

- Heterogeneous or patchy enhancement;
- Substantial paraspinal soft tissue involvement;
- Relative preservation of the intervertebral disc in early stages.

Accurate differentiation requires a high index of suspicion and early MRI acquisition, particularly in immunocompromised or TB-endemic populations. Optimal diagnosis should incorporate imaging characteristics, laboratory markers (e.g., interferon-gamma release assay, AFB stain), and histopathology or PCR when needed [20,21].

(2) Computed Tomography (CT)

CT is valuable for visualizing cortical erosion and bony destruction and for preoperative planning. It also provides a reliable assessment of instrumentation integrity and fusion status postoperatively.

(3) Plain Radiography

Although insensitive in early-stage disease, plain radiographs can reveal chronic osseous changes, alignment abnormalities, and hardware positioning.

(4) Nuclear Imaging

Radionuclide scans such as technetium-99m bone scintigraphy and gallium-67 imaging may be helpful when MRI is contraindicated or unavailable. These modalities offer functional insight into metabolic activity, but lack detailed anatomical resolution.

(5) Positron Emission Tomography/Computed Tomography (PET/CT)

Fluorodeoxyglucose-PET/CT (FDG-PET/CT) has emerged as a valuable imaging modality in the diagnosis and follow-up of spinal infections, including CPS. PET-CT is particularly useful in cases with the following:

- Inconclusive MRI findings;
- Persistent symptoms despite antibiotic therapy;
- Postoperative spine where hardware causes MRI artifacts;
- Suspected recurrence or systemic seeding.

Compared to conventional MRI, FDG-PET/CT provides superior sensitivity for detecting metabolically active infection, and its functional imaging capability aids in monitoring therapeutic response. Moreover, it can differentiate infection from sterile inflammation or neoplastic lesions. Recent studies support its complementary use alongside MRI in complex or refractory cases of CPS [22,23].

## 6. Treatment Strategies

The management of CPS poses considerable challenges due to its rarity, the complex anatomy of the cervical region, and the high potential for morbidity. An individualized treatment approach is essential, balancing conservative and surgical options based on the presence of neurological deficits, spinal instability, extent of infection, underlying comorbidities, and response to initial therapy [24,25,26,27,28,29].

### 6.1. Conservative Management

#### 6.1.1. Indications

Non-operative management is appropriate for patients with localized infection, preserved spinal stability, and no neurological deficits. It is most effective during early disease, particularly when infection is confined to the intervertebral disc or vertebral body without epidural abscess or spinal cord compression. Success requires accurate diagnosis, pathogen-specific antibiotic therapy, and close clinical monitoring [24,25].

#### 6.1.2. Antibiotic Therapy

Antibiotics are the cornerstone of conservative management and must be tailored to the causative organism. Treatment typically begins with an empirical regimen, followed by culture-guided therapy (Table 3) [30,31].

(1) Empirical Therapy

Empirical antibiotics should be initiated upon clinical suspicion of infection. Regimens should provide broad-spectrum coverage against Staphylococcus aureus (including MRSA) and Gram-negative organisms. A common approach includes vancomycin combined with a third-generation cephalosporin or piperacillin-tazobactam.

(2) Pathogen-Specific Therapy

Antibiotic selection should be revised accordingly once culture and sensitivity results are available. Treatment duration typically ranges from 6 to 12 weeks, with 4 to 6 weeks of intravenous therapy, followed by oral agents depending on clinical response.

(3) Transition to Oral Therapy

Transition to oral antibiotics may be considered in patients with clinical improvement and normalization of inflammatory markers (CRP, ESR). Criteria include resolution of fever, reduced pain, stable imaging findings, and absence of new neurological symptoms.

Continuous monitoring is essential. Treatment failure or worsening condition warrants reassessment and possible surgical referral.

#### 6.1.3. Immobilization

External immobilization serves as an important adjunct when surgery is contraindicated or delayed. It aims to minimize spinal motion, reduce mechanical stress, alleviate pain, and facilitate healing [26].

(1) Rigid Cervical Collars

These provide moderate immobilization and are typically used for 6 to 12 weeks in patients without instability or neurological compromise.

(2) Halo-Vest Immobilization

Used in cases of significant instability or when surgery is not feasible. It offers maximum immobilization by anchoring the head to the thorax. Application is generally maintained for 6 to 12 weeks, followed by gradual weaning based on radiologic and clinical improvement.

#### 6.1.4. Clinical Monitoring

Ongoing evaluation is essential to assess treatment response and detect complications early [27,28,29].

(1) Clinical Assessment

This includes the regular evaluation of pain severity, neurologic function, and general health status. The emergence of new or worsening neurological symptoms warrants immediate reassessment.

(2) Imaging Follow-up

MRI is preferred for monitoring soft tissue infection and abscess resolution, while CT is useful for evaluating bony healing and alignment.

(3) Laboratory Markers

Serial CRP and ESR are used to track inflammatory activity. Persistent or rising values may signal treatment failure or unresolved infection.

### 6.2. Surgical Management

Early surgical intervention has improved neurological outcomes, optimized infection control, and facilitated durable stabilization. Decision making should integrate clinical, radiographic, and patient-specific factors.

#### 6.2.1. Indications

Surgical intervention is indicated under the following conditions [24,25,26,27,28,29].

(1) Neurological Deficits

Progressive myelopathy, radiculopathy, or paralysis due to compression from abscesses, granulation tissue, or vertebral collapse.

(2) Spinal Instability

Instability from the vertebral body disc destruction or posterior element compromise, often presenting with kyphosis or subluxation.

(3) Extensive Abscess Formation

Large epidural, paravertebral, or retropharyngeal abscesses are unresponsive to medical therapy, especially when causing mass effects on the spinal cord or airway.

(4) Failure of Medical Therapy

Persistent pain, systemic signs, or radiologic progression despite adequate antibiotic treatment.

Table 4 provides a treatment algorithm for CPS based on neurologic status, stability, and abscess presence.

#### 6.2.2. Surgical Approaches

The surgical approach must be customized based on the location of the infection, vertebral destruction, neurological status, and patient comorbidities (Table 5).

(1) Anterior Cervical Discectomy and Fusion (ACDF)

**Figure 3:**
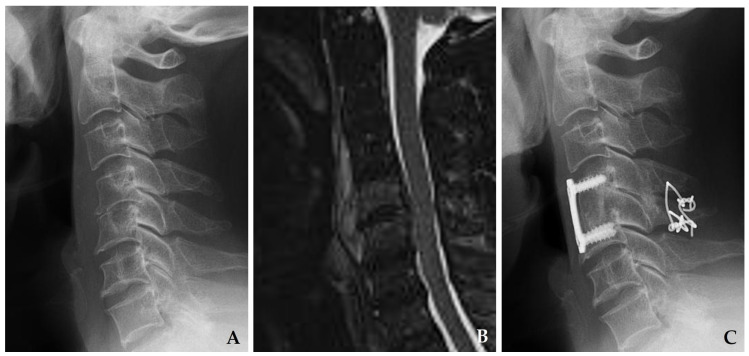
Initial plain radiograph (**A**) and magnetic resonance imaging (**B**) demonstrate disc space narrowing at C4–C5 with retropharyngeal and epidural abscesses. The patient underwent C4–C5 discectomy with autograft, anterior plating, and posterior wiring (**C**), achieving solid fusion and complete resolution of spondylodiscitis at one year postoperatively.

Preferred for 1–2-level disc space infections without significant vertebral body collapse. Allows for targeted debridement, interbody reconstruction, and early stabilization (Figure 3) [32,33].

(2) Anterior Cervical Corpectomy and Fusion (ACCF)

**Figure 4:**
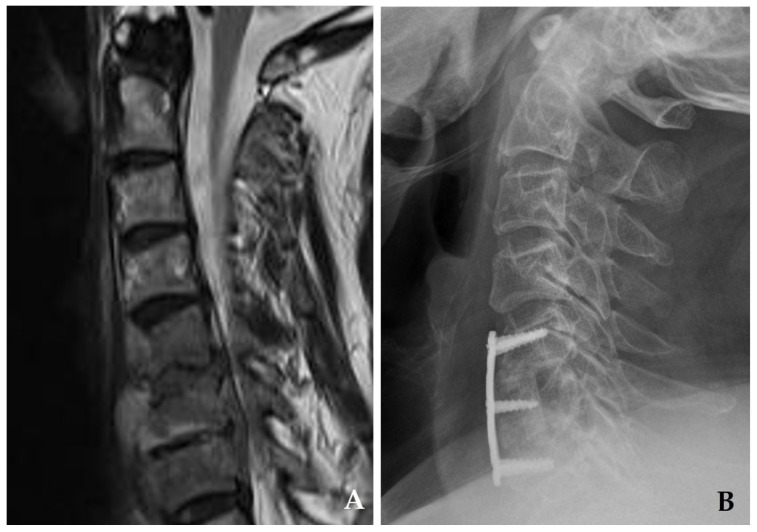
Initial magnetic resonance imaging (**A**) demonstrates disc space narrowing at C5–C6 and destructive changes in the C6 vertebral body with retropharyngeal and epidural abscesses. The patient underwent C5–C7 corpectomy with autograft and anterior plating (**B**), achieving solid fusion and complete resolution of spondylodiscitis at six months postoperatively.

Indicated for anterior abscesses, multilevel involvement, or vertebral body destruction. Enables wide decompression and realignment, though associated with higher technical complexity and graft-related complications (Figure 4) [32,33].

(3) Posterior Cervical Decompression and Fusion (PCDF)

This is employed for posterior element infections, dorsal epidural abscesses, or when anterior access is limited. It provides robust posterior stabilization but does not directly address anterior pathology [34,35,36].

(4) Combined Anterior–Posterior Approach (CAPA)

**Figure 5:**
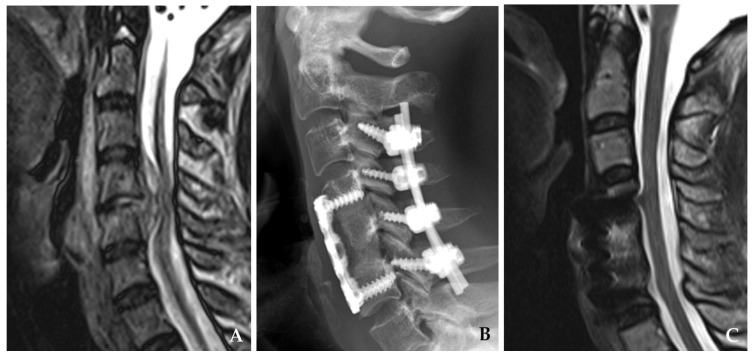
Initial magnetic resonance imaging (**A**) demonstrates disc space narrowing at C4–C5 and a large epidural abscess causing spinal cord compression. The patient underwent C4–C6 corpectomy with autograft and anterior plating (**B**), achieving solid fusion and complete resolution of spondylodiscitis at six months postoperatively (**C**).

Necessary for extensive two-column disease, severe kyphosis, or multilevel vertebral collapse. While associated with greater operative morbidity, it provides comprehensive decompression and 360° stabilization, making it the optimal choice for complex or refractory infections (Figure 5) [37,38].

#### 6.2.3. Anterior Plating Versus Non-Plating in ACDF and ACCF

**Figure 6:**
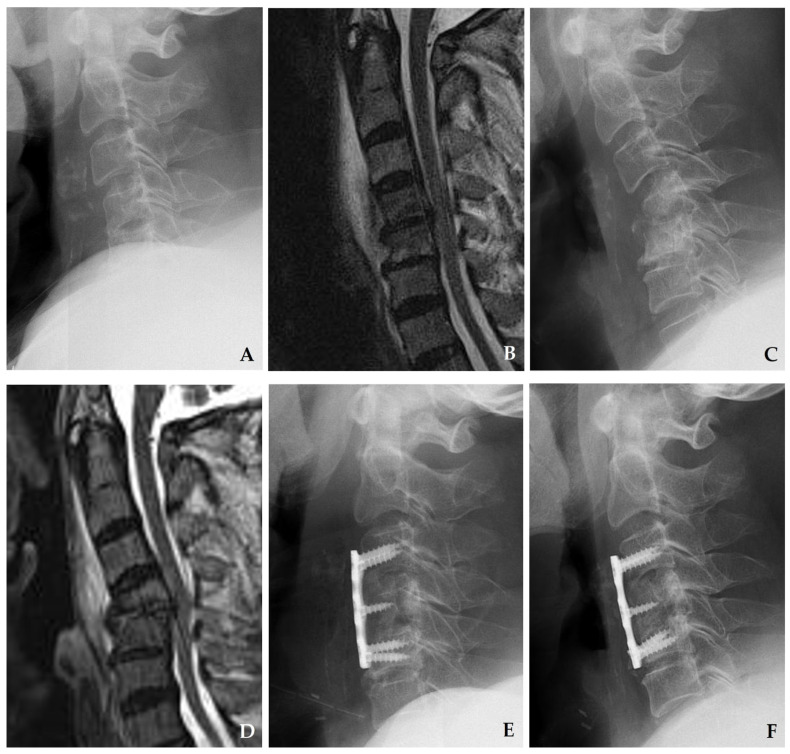
Initial plain radiograph (**A**) and magnetic resonance imaging (MRI) (**B**) demonstrate disc space narrowing at C4–C5 with a retropharyngeal abscess. After one month of antibiotic treatment, follow-up plain radiograph (**C**) and MRI (**D**) reveal progressive destructive changes in the C4–C5 vertebral bodies with kyphotic deformity, accompanied by retropharyngeal and epidural abscesses compressing the spinal cord. The patient underwent C3–C5 corpectomy with autograft and anterior plating (**E**), achieving solid fusion and complete resolution of spondylodiscitis at one year postoperatively (**F**).

**Figure 7:**
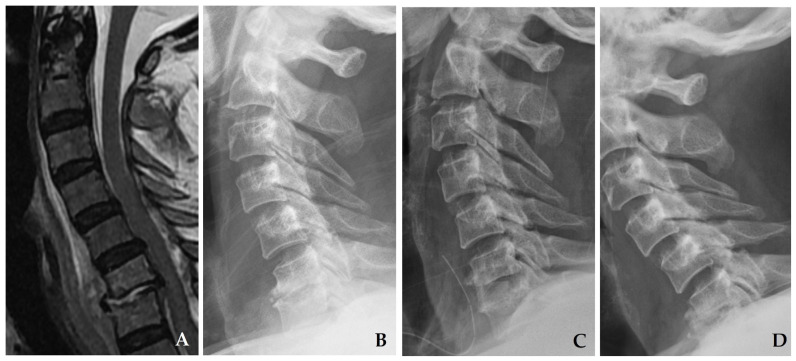
Initial magnetic resonance imaging (**A**) and plain radiograph (**B**) demonstrate disc space narrowing at C6–C7 with a retropharyngeal abscess. The patient underwent C6–C7 discectomy with autograft without anterior plating (**C**); however, collapse of the autograft and development of kyphosis were observed at two months postoperatively (**D**).

The use of anterior plating and graft material selection in CPS remains an area of ongoing debate. Proponents of anterior plating argue that it enhances biomechanical stability, facilitates early mobilization, and reduces the risk of graft subsidence, especially in cases with compromised endplates or multilevel involvement [39,40]. Several studies report higher fusion rates and lower failure rates with instrumentation, even in infected settings, when combined with meticulous debridement and pathogen-specific antibiotic therapy (Figure 6) compared to non-plating in ACDF and ACCF (Figure 7).

Conversely, other reports caution against routine anterior plating due to the theoretical risk of bacterial colonization on hardware and the formation of biofilms, which may contribute to persistent or recurrent infection. These concerns have led some authors to advocate for non-instrumented fusion, particularly in patients with limited segmental destruction, good bone quality, or early-stage disease.

#### 6.2.4. Interbody Graft Materials

The choice of graft material—autograft, allograft, or interbody cages (PEEK/titanium)—adds further complexity. Autologous tricortical iliac crest grafts remain the gold standard in many centers due to superior osteoinductive and osteoconductive properties. However, donor site morbidity and prolonged operative time are notable drawbacks. Allografts and PEEK cages offer logistical advantages and reduced morbidity but have been associated with higher rates of graft resorption and pseudarthrosis in some series. Titanium cages provide immediate structural stability but may obscure postoperative imaging and are less forgiving in osteomyelitic bone [39,41,42,43,44,45,46,47,48,49,50,51]. Optimal graft selection must balance mechanical stability, biological compatibility, and infection control. In CPS, where infection eradication, spinal realignment, and long-term stability are critical, the careful selection of graft material is essential for surgical success and recurrence prevention [52,53]. Table 6 presents a detailed comparison of interbody graft materials, including their mechanical properties, biological characteristics, fusion potential, and associated risks.

#### 6.2.5. Biologic Adjuncts to Interbody Fusion: Bone Morphogenetic Protein and Demineralized Bone Matrix

Biologic adjuncts such as bone morphogenetic protein (BMP) and demineralized bone matrix (DBM) have enhanced spinal fusion, particularly in revision procedures or biologically compromised patients with impaired osteogenesis. However, their use in the setting of active spinal infection remains controversial due to concerns regarding safety and efficacy [54,55]. Table 7 presents a comparative overview of BMP and DBM as biologic adjuncts.

While BMP and DBM can enhance fusion in appropriately selected cases, their routine use during active spinal infection is not recommended. These biologics may be considered in the following settings:

- Delayed reconstruction following infection eradication;
- Well-controlled infectious environments;
- Revision surgeries requiring biological augmentation provided that stable fixation and thorough debridement have been achieved.

Their application should be judicious, guided by an individualized risk–benefit assessment, and undertaken in consultation with surgical and infectious disease teams.

#### 6.2.6. Posterior Surgical Techniques

Posterior cervical decompression and fusion (PCDF) is a critical option in the surgical management of CPS, particularly when infection involves the posterior elements or dorsal epidural space, or anterior access is contraindicated due to anatomical or pathological limitations [56,57]. Primary indications include the following:

- Involvement of posterior bony or ligamentous structures (e.g., laminae, facet joints) with radiologic or intraoperative signs of infection or destruction;
- Dorsally located epidural abscesses causing significant spinal cord or nerve root compression;
- Multilevel instability or kyphosis requiring supplemental posterior stabilization;
- Anterior contraindications, including prior anterior surgery, dense scar formation, or vascular anomalies.

PCDF typically involves multilevel laminectomy for neural decompression and abscess drainage, followed by posterior fixation using lateral mass screws (C3–C6) or pedicle screws (C7–T1), depending on anatomical and biomechanical considerations. Posterior fixation provides robust three-column stabilization, making it suitable for long-segment constructs or cases with insufficient anterior support. PCDF is frequently combined with anterior approaches in complex infections to achieve circumferential decompression and fusion.

#### 6.2.7. Combined Surgical Techniques

Combined anterior–posterior approaches (CAPA) are indicated in complex or extensive CPS, particularly when infection spans both the anterior and posterior spinal columns or when single-approach procedures fail to achieve adequate debridement, decompression, or stabilization [37,38,55,56]. Indications include the following:

- Multilevel or circumferential infection;
- Severe kyphotic deformity;
- Extensive bony destruction with mechanical instability;
- Incomplete debridement or decompression via a single approach.

CAPA enables comprehensive infection control, incorporating anterior debridement and reconstruction (ACDF or ACCF with plating) followed by posterior laminectomy and instrumentation. This strategy corrects deformity, provides circumferential stabilization, and enhances fusion success.

Although associated with longer operative times, greater blood loss, and increased perioperative risk, CAPA offers superior infection clearance and biomechanical reconstruction in refractory CPS. Successful outcomes depend on meticulous preoperative planning, multidisciplinary input, and precise surgical execution, whether staged or simultaneous.

#### 6.2.8. Instrumentation Considerations

Instrumentation is crucial for restoring stability, especially in cases with vertebral collapse, multilevel disease, or deformity. Spinal implants do not increase infection risk when implemented appropriately, provided that thorough debridement and targeted antimicrobial therapy are performed [43,58,59].

- Anterior instrumentation, including plating, is considered safe in infected environments and offers several benefits:
  ○ Preservation of cervical alignment;
  ○ Prevention of graft subsidence or migration;
  ○ Enhancement of fusion success.
- Posterior-only instrumentation may be necessary in cases where the following are true:
  ○ The anterior column is severely compromised or contaminated;
  ○ Anterior plating is contraindicated (e.g., poor bone quality, extensive scar tissue);
  ○ Additional posterior decompression is needed.

Posterior fixation provides powerful mechanical support and can be used alone or with anterior constructs to achieve complete circumferential stability.

#### 6.2.9. Postoperative Care

Comprehensive postoperative management is essential for CPS infection control, structural healing, and functional recovery. Key components include the following:

- Antibiotic Therapy

Administer intravenous antibiotics for 4–6 weeks, followed by oral therapy to complete a 6–12-week course, adjusted based on the identified organism, clinical response, and inflammatory markers [30,31].

- Immobilization

Apply rigid cervical collars or halo-vest orthoses for 6–12 weeks, depending on construct stability and extent of decompression/fusion [24,25].

- Follow-Up Imaging
  ○ MRI: Preferred for tracking soft tissue response, abscess regression, and residual infection.
  ○ CT: Ideal for assessing graft incorporation, bony healing, and implant integrity.
- Laboratory Monitoring
  ○ Monitor CRP and ESR weekly during IV therapy.
  ○ Monitor biweekly during the oral antibiotic phase.

Postoperative care should be multidisciplinary, involving infectious disease specialists, radiologists, rehabilitation teams, and the spine surgeon. Long-term follow-up is necessary to detect recurrence, pseudarthrosis, or delayed complications such as implant failure or adjacent segment disease.

## 7. Evidence-Based Outcomes and Prognosis

Outcomes following treatment for cervical pyogenic spondylitis (CPS) are variable and depend on a range of interrelated factors. While favorable results have been reported in cases treated with timely diagnosis, appropriate antimicrobial therapy, and well-executed surgical intervention, the prognosis cannot be universally considered optimistic. Several studies underscore the importance of early diagnosis and intervention as the most consistent predictor of neurological recovery and infection control. Delayed treatment is associated with increased risk of irreversible neurological damage, progression to spinal instability, and systemic complications, including sepsis [60,61,62,63,64,65]. Based on the current literature, the following prognostic factors are supported by varying levels of evidence.

### 7.1. Factors Strongly Associated with Favorable Prognosis

(1) Early initiation of pathogen-directed antibiotic therapy (within 48–72 h of symptom onset).
(2) Absence of baseline neurological deficit or only mild deficits at presentation.
(3) Localized infection without epidural abscess or extensive vertebral destruction.
(4) Use of surgical debridement in cases with instability or abscess compression, particularly when decompression is combined with effective antimicrobial coverage.

### 7.2. Factors Associated with Poor Prognosis or Higher Complication Rate

(1) Delayed diagnosis (>2 weeks from symptom onset).
(2) Presence of severe or progressive neurological deficits (e.g., myelopathy, bowel/bladder dysfunction).
(3) Multilevel involvement and vertebral body collapse leading to structural instability.
(4) Immunocompromised host status, including uncontrolled diabetes mellitus, chronic renal disease, and steroid dependency.
(5) Infection with high-virulence organisms, especially MRSA or Gram-negative bacilli.

### 7.3. Inconclusive Conflicting Evidence

(1) The impact of plating versus non-plating on long-term recurrence and functional outcome remains debated due to the lack of prospective studies.
(2) The choice of graft material (autograft vs. cage vs. allograft) has shown variable results across small-scale case series, with no high-level comparative data available.

While many patients achieve infection resolution and functional recovery, the prognosis must be viewed through a cautious and individualized lens. Prognostic estimation should consider pathogen virulence, neurological status, structural stability, and host immunity. Prospective multicenter data are urgently needed to establish risk stratification models and outcome prediction tools in CPS.

## 8. Complications and Their Management

CPS is associated with a range of complications, some of which carry significant morbidity and require prompt recognition and intervention. These include persistent or recurrent infection, nonunion (pseudarthrosis), neurological deterioration, and postoperative surgical complications. Optimal management relies on early recognition, evidence-based interventions, and rigorous perioperative and postoperative monitoring (Table 8) [60,66,67,68,69,70].

### 8.1. Persistent or Recurrent Infection

Persistent or recurrent infection may result from incomplete debridement, suboptimal antimicrobial therapy, or biofilm formation on implants. Biofilms impair immune clearance and hinder antibiotic penetration, often necessitating prolonged treatment and possible revision surgery. Management strategies include the following:

- Culture-directed intravenous antibiotics, followed by a prolonged oral regimen;
- Revision surgery with hardware removal, repeat debridement, and staged or immediate re-instrumentation in confirmed implant-related infection;
- Long-term suppressive antibiotic therapy for high-risk patients for whom eradication is not feasible.

Monitoring should involve serial inflammatory markers (CRP, ESR) and repeat imaging (MRI or CT) to assess therapeutic response and detect occult infection. Although recurrence is rare with appropriate management, unresolved infection increases the risk of chronic morbidity, instability, and neurological decline.

### 8.2. Nonunion or Graft Failure

Nonunion, or spinal fusion failure, is a notable complication, particularly in infection-induced bone resorption or mechanical instability. Fusion failure is more common in non-instrumented procedures due to inadequate stabilization. Contributing factors include the following:

- Persistent infection or incomplete debridement;
- Use of non-osteogenic or poorly integrated graft materials;
- Suboptimal surgical technique or graft placement;
- Insufficient postoperative immobilization.

Clinical signs may include persistent axial pain, radiologic pseudarthrosis, or progressive deformity. Management typically involves revision surgery, employing rigid anterior and/or posterior instrumentation, improved graft materials (e.g., autograft, titanium cage, biologics), and strict immobilization protocols. Prevention requires meticulous surgical technique, stable fixation, and adequate infection control.

### 8.3. Neurological Deterioration

Neurological deterioration is among the most serious complications of CPS. It may result from the following:

- Unrelieved neural compression due to abscess, vertebral collapse, or inflammatory tissue;
- Inadequate decompression or failure to stabilize the spine.

Prompt decompression and stabilization are essential to minimize irreversible neurological injury. Early surgical intervention, particularly in progressive myelopathy or radiculopathy cases, correlates with improved outcomes. Intraoperative neuromonitoring may enhance safety during high-risk procedures. Postoperative neurological evaluation is critical, and any new or worsening deficits require urgent imaging and potential revision surgery. Delays in intervention are strongly associated with poorer neurologic recovery.

### 8.4. Postoperative Complications

Several surgery-related complications may occur and require prompt identification and treatment.

(1) Wound Infection

It may result from intraoperative contamination or persistent infection. If necessary, management includes empirical and culture-specific antibiotics, wound irrigation, and surgical debridement.

(2) Cerebrospinal Fluid (CSF) Leak

It is usually due to dural injury during decompression. Small leaks may resolve with bed rest or lumbar drainage, but persistent leaks often require surgical repair.

(3) Hematoma Formation

It can result in acute neurological decline from the mass effect. Requires emergent evacuation in symptomatic cases.

(4) Graft Subsidence or Collapse

It is more common in non-instrumented constructs or with poor endplate preparation. Prevention includes appropriate graft selection, endplate preservation, and anterior or posterior instrumentation for stabilization.

Postoperative monitoring protocols should include the following:

- Serial MRI and CT to evaluate graft integrity, spinal alignment, and infection resolution;
- Regular CRP and ESR monitoring during both intravenous and oral antibiotic phases;
- Multidisciplinary coordination with infectious disease, radiology, and rehabilitation teams.

Proactive complication management and structured follow-up protocols are essential to maximize outcomes and minimize reoperation rates in this vulnerable patient population.

## 9. Conclusions

CPS is a rare but potentially debilitating spinal infection that requires early recognition and tailored multidisciplinary management to prevent irreversible neurological compromise and spinal deformity. Although accumulating clinical experience has improved diagnostic accuracy and surgical outcomes, the current body of evidence remains largely retrospective, single-centered, and heterogeneous in methodology. This review has several inherent limitations, including reliance on low- to moderate-quality studies, lack of high-level prospective data, and variability in surgical indications, graft choices, and antibiotic protocols across institutions. These limitations restrict the generalizability of existing recommendations and underscore the need for further rigorous investigation.

To address these gaps, we propose the following priority areas for future research:

1. Multicenter prospective cohort studies and randomized controlled trials (RCTs) to determine the comparative efficacy of anterior plating versus non-plating in infected fields.
2. Standardization and outcome comparison of graft materials (autograft, titanium cage, PEEK, allograft) specifically in the context of spinal infection.
3. Definition of optimal duration and route (IV vs. oral) of antibiotic therapy, guided by biomarkers and imaging criteria for infection resolution.
4. Development of a risk stratification model to guide surgical decision making based on neurological status, spinal stability, and host immune function.
5. Long-term follow-up data collection on spinal alignment, fusion success, reinfection rates, and health-related quality of life in surgically versus conservatively treated patients.

Moving forward, collaborative efforts are essential to generate high-quality evidence and establish evidence-based guidelines that can inform individualized treatment plans for CPS across diverse clinical settings.

## Figures and Tables

**Table 1:** Microbiology of cervical pyogenic spondylitis.

Category	Pathogen	Clinical Features and Associations
Common Pathogens	*Staphylococcus aureus* (60–70%) (MSSA, MRSA)	MSSA and MRSA strains MRSA associated with aggressive disease, epidural abscess, vertebral collapse
	*Streptococcus* species (*S. viridans*, Group B)	Associated with endocarditis and dental procedures Hematogenous spread causing discitis, osteomyelitis
	Gram-negative bacilli (*E. coli*, *Klebsiella pneumoniae*, *Pseudomonas aeruginosa*, *Proteus* spp.)	Common in immunocompromised patients Linked with UTIs, intravenous drug use, surgical complications Biofilm formation on hardware
	Anaerobic bacteria (e.g., *Prevotella melaninogenica*)	Rare; related to oropharyngeal infections/abscesses extending to cervical spine
Atypical Pathogens	*Mycobacterium tuberculosis* (TB)	Indolent presentation (low-grade fever, night sweats, weight loss) Mildly elevated inflammatory markers
	Fungal pathogens (*Candida*, *Aspergillus* spp.)	Rare; immunocompromised individuals (HIV, post-transplant, immunosuppression) Nonspecific clinical features

**Table 2:** Comparison of clinical features: cervical versus thoracolumbar pyogenic spondylitis.

Features	Cervical Spine	Thoracolumbar Spine
Pain Location	Axial neck pain, occipital radiation	Back pain, flank pain
Neurologic Involvement	Early and severe (cord compression, upper limb deficits, quadriparesis)	Often delayed or localized (e.g., cauda equain syndrome)
Myelopathic Signs	Common (gait disturbance, spasticity)	Less common
Unique Symptoms	Dysphagia, hoarseness, occipital neuralgia, drop attack	Radicular pain, paraspinal spasm
Cranial Proximity Symptoms	Possible (C1–C2 lesions: headache, vertigo)	None
Fever and Systemic Signs	Often absent or subtle	More common
Prevertebral Soft Tissue Swelling	Frequent (may cause airway symptoms)	Rare
Diagnostic Delay Risk	High due to atypical or silent onset	Moderate

**Table 3:** Empirical and pathogen-specific antibiotic regimes.

Antibiotic Regimen	Indication	Example Agents	Title 4
Empirical IV Therapy	Initiation before culture results	Vancomycin + Ceftriaxone or Piperacillin, Tazobactam	4–6 weeks (IV), then reassess
MSSA	Culture-confirmed MSSA	Nafcillin or Cefazolin	IV for 4–6 weeks
MRSA	Culture-confirmed MRSA	Vancomycin or Daptomycin	IV for 6–8 weeks
Gram-Negative Bacilli	Immunocompromised or UTI-related	Cefepime, Meropenem, or Ciprofloxacin	IV for 4–6 weeks, then oral if stable
TB or Atypical Mycobacterial	Suspected TB (endemic region)	RIPE therapy (Rifampin, INH, Pyrazinamide, Ethambutol)	≥6–9 months
Fungal Infection	Immunocompromised patients	Amphotericin B, Fluconazole	prolonged therapy, tailored

**Table 4:** Treatment algorithm for CPS based on neurologic status, stability, and abscess presence.

Neurologic Status	Stability	Abscess	Recommend Treatment
Intact	Stable	None	IV antibiotics only, monitor closely
Intact	Stable	Small	IV antibiotics + possible aspiration
Intact	Unstable	±Any	Anterior/posterior surgery + antibiotics
Mild Deficit	Any	Present	Urgent anterior surgery ± posterior support + antibiotics
Severe Deficit	Any	Present	Emergent decompression (anterior or combined) + antibiotics

**Table 5:** Summary of surgical approaches for cervical pyogenic spondylitis.

Approach	Indications	Surgical Technique/Advantages/Limitations
Anterior (ACDF)	Localized disc space infection, 1–2 levels, without vertebral collapse	Discectomy, debridement, interbody grafting, anterior plating Less invasive, preserves vertebral bodies, promotes early fusion Limited for vertebral body involvement, risk of incomplete debridement
Anterior (ACCF)	Vertebral body destruction, anterior abscess, multilevel disease	Corpectomy, graft placement, anterior plating Better decompression for anterior abscesses, restores alignment Technically demanding, higher risk of dysphagia and graft complications
Posterior (PCDF)	Posterior element involvement, dorsal abscess, or anterior contraindications	Laminectomy, abscess drainage, lateral mass, or pedicle screw fixation Effective for dorsal abscesses, supports multilevel stability May not address anterior disease; possible need for a combination
Combined (CAPA)	Extensive/multilevel disease involving both columns, kyphosis, instability	Anterior reconstruction (ACDF/ACCF) + posterior decompression and fixation 360° decompression and stabilization, superior mechanical support Higher morbidity requires careful planning and longer recovery

**Table 6:** Interbody graft materials in cervical pyogenic spondylitis surgery.

Graft Material	Key Features	Indications/Contraindications
Autograft (ICBG)	Osteogenic, osteoinductive, and osteoconductive properties	Gold standard for infected spine cases; used when optimal fusion is critical Severe osteoporosis, poor donor site condition Donor site pain, infection, fracture at harvest site
PEEK Cage	Radiolucent inert polymer; used with bone graft or substitutes	Common in degenerative and infectious spine cases when paired with autograft Severe active infection without proper debridement Nonunion if not combined with graft; possible subsidence
Titanium Cage	Biocompatible metal with osseointegration capacity; strong mechanical support	Useful in infected spine with vertebral collapse requiring strong support Limited use in patients with metal allergy or inferior bone quality Cage subsidence, artifact on imaging, infection persistence
Allograft	Donor bone from a cadaver, sterilized; lacks living cells	Rarely recommended in infection due to poor incorporation Active infection, high risk of graft rejection Nonunion, graft resorption, delayed fusion

**Table 7:** Biologic adjuncts: bone morphogenetic protein and demineralized bone matrix.

Biologic Adjuncts	Key Features/Indications/Contraindications	Advantages/Disadvantages/Complications
BMP	Osteoinductive cytokines promote bone growth Used when autograft quantity is insufficient or revision surgery Infection, malignancy history, pregnancy	Enhances fusion, especially in difficult cases; avoids harvesting autograft Costly; potential for ectopic bone formation and inflammation Inflammation, radiculitis, heterotopic ossification
DBM	Processed allograft containing collagen and growth factors Adjunct in fusion when combined with other grafts Infection site with poor debridement; not a standalone structural graft	Some osteoinductive activity; often used with autograft or PEEK Weaker mechanical support; variable biological activity Incomplete fusion; immunologic response

**Table 8:** Common complications and management strategies in CPS.

Complications	Clinical Significance	Management Strategies
Persistent/Recurrent Infection	May lead to revision surgery	Repeat debridement, adjust antibiotics, consider implant removal
Nonunion/Pseudoarthrosis	Causes pain, deformity	Revision surgery with rigid fixation and biologic augmentation
Neurologic Deterioration	Risk of irreversible Deficits	Prompt decompression and stabilization
Graft Subsidence/ Collapse	Leads to kyphosis or instability	Use of plated constructs Careful endplate preparation
CSF Leak	Delayed healing, infection risk	Conservative or surgical repair, lumbar drainage
Wound Infection	Delayed healing or recurrence	Wound care, targeted antibiotics, possible reoperation
Hardware Complications	Screw loosening, dysphagia	Monitor or revise implant depending on severity

## Data Availability

Not applicable.

## Abbreviations

The following abbreviations are used in this manuscript:

MSSA	Methicillin-Sensitive *Staphylococcus aureus*
MRSA	Methicillin-Resistant *Staphylococcus aureus*
TB	Mycobacterium Tuberculosis
PCR	Polymerase Chain Reaction
PEEK	Polyetheretrherketone
ICBG	Iliac Crest Bone Graft
BMP	Bone Morphogenic Protein
DBM	Demineralized Bone Matrix
MRI	Magnetic Resonance Imaging
CT	Computed Tomography
ACDF	Anterior Cervical Discectomy and Fusion
ACCF	Anterior Cervical Corpectomy and Fusion
PCDF	Posterior Cervical Decompression and Fusion
CAPA	Combined Anterior–Posterior Approaches
